# The Glycosylation of AGP and Its Associations with the Binding to Methadone

**DOI:** 10.1155/2013/108902

**Published:** 2013-07-15

**Authors:** Jennifer L. Behan, Yvonne E. Cruickshank, Gerri Matthews-Smith, Malcolm Bruce, Kevin D. Smith

**Affiliations:** ^1^School of Life, Sport and Social Sciences, Edinburgh Napier University, Sighthill Campus, Edinburgh EH11 4BN, UK; ^2^School of Nursing, Midwifery and Social Care, Edinburgh Napier University, Edinburgh EH11 4BN, UK; ^3^Community Drug Problem Service, Spittal Street Centre, Edinburgh EH3 9DU, UK

## Abstract

Methadone remains the most common form of pharmacological therapy for opioid dependence; however, there is a lack of explanation for the reports of its relatively low success rate in achieving complete abstinence. One hypothesis is that *in vivo* binding of methadone to the plasma glycoprotein alpha-1-acid glycoprotein (AGP), to a degree dependent on the molecular structure, may render the drug inactive. This study sought to determine whether alterations present in the glycosylation pattern of AGP in patients undergoing various stages of methadone therapy (titration < two weeks, harm reduction < one year, long-term > one and a half years) could affect the affinity of the glycoprotein to bind methadone. The composition of AGP glycosylation was determined using high pH anion exchange chromatography (HPAEC) and intrinsic fluorescence analysed to determine the extent of binding to methadone. The monosaccharides galactose and N-acetyl-glucosamine were elevated in all methadone treatment groups indicating alterations in AGP glycosylation. AGP from all patients receiving methadone therapy exhibited a greater degree of binding than the normal population. This suggests that analysing the glycosylation of AGP in patients receiving methadone may aid in determining whether the therapy is likely to be effective.

## 1. Introduction

It has been reported that the number of people who could be considered as being involved in opioid abuse, commonly coadministered with the benzodiazepines (BDZs), is between 12.8 and 21.8 million individuals [1]. Opioid dependence represents a universal problem to society and methadone remains as the most common form of pharmacological therapy. Similar to other drugs, the desired action of methadone requires that it must attain a certain *in vivo* concentration (the minimally effective concentration, MEC) at its site of action in order to provide a therapeutic effect. Unfortunately the success rate of methadone in achieving complete abstinence is poor and reportedly as low as 7% [2].

According to the free drug hypothesis, only a drug which is in a free unbound state *in vivo* is active. As most drugs access their target site of action through the bloodstream, they come into contact with numerous plasma proteins including albumin and *α*-1-acid glycoprotein (AGP), which are capable of binding the drug and rendering it inactive. After administration, methadone is strongly associated with AGP [3], therefore, alterations in the level and structure of the protein could greatly affect the efficacy of the drug. When doses of methadone are below the MEC, withdrawal symptoms may arise, including nausea, anxiety, tremors and dissipation of the euphoric “high” [4, 5]. Conversely, if concentrations exceed the threshold too rapidly or by too much, the effects of an overdose are displayed.

During the acute phase response to infection and disease, levels of AGP are known to increase two- to fivefold [6]. The increased production of AGP and any other drug binding plasma proteins causes an immediate shift in the binding equilibrium between drug and proteins upon the administration of the drug [7]. Consequently, the plasma level of bioactive (unbound) drug available to the target site of action or receptor is reduced, alongside its efficacy [8]. Rostami-Hodjegan and colleagues [9] noted that in heroin-dependent individuals with signs of opiate withdrawal, levels of AGP were elevated and were thought to reduce the level of active unbound methadone, promoting withdrawal symptoms.

Like many hepatically synthesised proteins, AGP is also modified through the addition of oligosaccharide or glycan chains (glycosylation). The process of glycosylation is an ordered and functionally significant process which results in a high degree of structural heterogeneity. The aforementioned variability is essentially a result of different cell types possessing a diverse array of enzymes thus catalysing a range of reactions to produce distinct glycans [10, 11]. AGP is extensively glycosylated (45%) with five oligosaccharide chains and exists *in vivo* as a heterogeneous population of variants (glycoforms) owing to differing occupancy of the five glycosylation sites by different oligosaccharide structures. Heterogeneity arises through subtle structural differences in monosaccharide sequence and linkages, degree of branching [bi-(2), tri-(3), tetra-(4) antennary], and the extent and number of charged sialic acid groups (sialylation). During several physiological and pathological conditions, the oligosaccharide “fingerprint” of AGP is altered [12, 13]. 

It is now widely accepted that AGP is an important drug-binding protein in the serum as it binds both endogenous and exogenous ligands. The drug-binding capabilities of AGP and the binding site of human AGP have been well characterised [14]. Muller [15] reported that AGP glycans are not critical in the interaction with drugs at the binding site, and instead it is solely dependent on the formation of the correct tertiary structure, describing the folding of the protein into its three-dimensional structure which is essentially determined by the sequence of amino acids (primary structure). However, although the drug binding site of AGP is peptide in nature, the hydrodynamic mass and surface location of the oligosaccharide chains are likely to affect the conformation, and correct folding, of both the protein in general and the binding site in particular. Therefore an alteration in the glycosylation of AGP is likely to affect the extent of binding to drugs.

This study sought to investigate whether differences in the relative level and glycosylation of AGP existed between that isolated from opioid-dependent individuals and a “normal” healthy population, which could correlate with variations in the binding to methadone. The structure of glycans expressed by AGP was analysed using high pH anion exchange chromatography (HPAEC). Subsequently, fluorescence quenching data was utilised as an indicator of binding to methadone.

## 2. Materials and Methods

### 2.1. Materials

Cibacron Blue 3GA, Dulbecco's phosphate-buffered saline, dimethyl sulphoxide Glacial acetic acid, (±)-methadone, polyethylene glycol (PEG) 3350, potassium chloride, potassium thiocyanate, Red Sepharose CL-6B, sodium acetate, sodium azide, sodium chloride, theophylline, and Trizma base were supplied by Sigma-Aldrich (Poole, UK). Ethanol was purchased from Bamford Laboratories Ltd., (Norden Rochdale, UK). HPLC-grade water was obtained from Rathburn Chemicals Ltd. (Walkerburn, UK). The poly prep disposable 10 mL columns were purchased from Bio-Rad, (Hemel Hempstead, UK). Amicon Ultra-4 centrifugal filter devices with a MW cutoff of 10,000 from Millipore (UK) Ltd. sodium hydroxide (50% w/v) were supplied by VWR International Ltd. (Lutterworth, UK). New England Biolabs Inc., (Hertfordshire, UK) provided the peptide-N-Glycosidase F (PNGase F) purified from *Flavobacterium meningosepticum*, 10% NP-40, and NE Buffer G7. HPAEC-PAD equipment, including the DX500 system and Carbopac PA-100 column, was purchased from Dionex, Camberley, UK. AGP N-linked glycan library was supplied by Prozyme (Europa Bioproducts Ltd., Cambridgeshire, UK). Nunc (Germany) supplied the 96-well microtitre plates, and A BMG Labtech Optima fluorimeter (Germany) was used to analyse the intrinsic fluorescence.

### 2.2. Methods

Ethical approval was granted by Lothian NHS and Edinburgh Napier University Ethics committees which allowed 2–5 mL blood samples to be obtained from consenting adults over 18 years of age (*n* = 20) undergoing various stages of treatment for opioid addictions at the Community Drug Problem Service (CDPS) in Edinburgh. Patient demographics are summarised in Table 1. Individuals were undergoing several phases including initial two-week titration (T), harm reduction (HR), or long-term methadone treatment (LT) and had no concomitant conditions present. Heparinised blood samples from “normal” healthy individuals were pooled by the blood transfusion service (BTS).

#### 2.2.1. Isolation of AGP from Plasma

AGP was isolated from all blood samples using protocols similar to those reported by Elliott et al. [16]. SDS-PAGE indicated that the two-column isolation technique generated relatively pure AGP. Salt introduced during the isolation phase was removed using centrifugal filter devices and HPLC-grade water. The levels of isolated AGP were determined spectrophotometrically. 

#### 2.2.2. Monosaccharide Analysis

Preparation of the glycans was similar to methods reported by Fan et al. [17]. AGP (50 *μ*g) was hydrolysed with 4 M HCl (six hours) and 2 M TFA (four hours) separately; we also found that the use of HCl rendered the neutral monosaccharides unstable over long periods of time. Also, the TFA was thought to be too weak to cleave N-acetylglucosamine (GlcNAc) residues from the core—as demonstrated by a higher level of GlcNAc in the presence of HCl compared to TFA. A Dowex cation-exchange resin was used to purify the neutral monosaccharides. HPAEC was used to separate and quantify the monosaccharide components. 

#### 2.2.3. Oligosaccharide Analysis

Structural analysis was also performed using HPAEC. Typically 100 *μ*g of AGP was denatured at 100°C for three hours and the dried sample then treated with a reaction mixture of HPLC-grade water (79% v/v), NE Buffer G7 (10% v/v), NP-40 (10% v/v), and PNGaseF (5 U). The mixtures were incubated at 37°C for 24 hours and then a further 5 U of PNGase F was added. After a final 24-hour incubation at 37°C, ethanol precipitation was performed at 3 : 1 ratio with the reaction mixture. The samples were then stored at −20°C overnight and analysed by HPAEC.

#### 2.2.4. Drug Binding Analysis

The binding of methadone to the isolated AGP preparations was investigated using intrinsic fluorescence studies at the microtitre level. Commercial AGP (5 mg/mL) was dissolved in d-PBS and plated in 10 *μ*L aliquots (*n* = 3). A range of theophylline (positive control) and methadone concentrations (0 *μ*M–2500 *μ*M) were added in 10 *μ*L volumes—having been dissolved in DMSO. A final reaction volume of 100 *μ*L was generated through the addition of D-PBS. Plates were excited at 280 nm and fluorescence emitted at 340 nm (AGP maximum emission) recorded. The experiment was repeated with AGP concentrations of 0.5 m/mL to allow for the low levels of AGP isolated from patient groups and ensures that the technique was adaptable to this level. Commercial and isolated AGP was plated (0.5 mg/mL) and methadone standards added to include values representing the therapeutic level of methadone (~4000 ng/mL) and others out-with this; all were diluted tenfold upon plating. 

#### 2.2.5. Statistical Analysis

Two sample *t*-tests and one-way ANOVAs (with Tukey's post hoc) were performed using Minitab, version 15, to determine the statistical significance of all quantitative data generated during the various analyses.

## 3. Results and Discussion

### 3.1. AGP Levels

**Figure 1:**
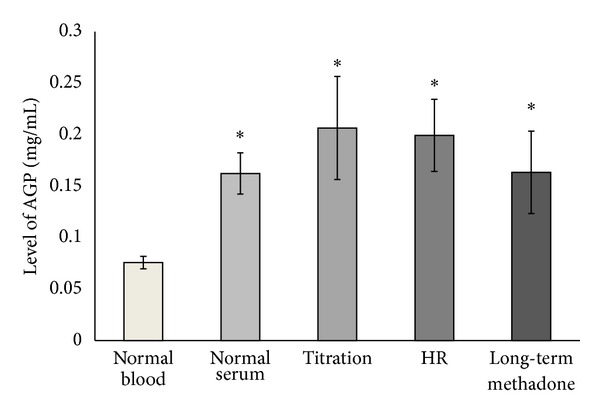
Relative level of AGP isolated from treatment groups. Summary of the mean level of AGP isolated from individuals at different stages of methadone therapy. *Statistically different to “normal” blood (*P* < 0.05).

Using a set of standards, an AGP calibration curve was generated and used to determine the relative level of AGP isolated from the individual samples (Figure 1). Statistical analysis was performed with two sample *t*-tests to compare the relative difference between each patient group and heparinised blood from a “normal” healthy population. Significantly lower levels of AGP were isolated from the “normal” blood population (*P* < 0.05) compared to those from all other treatment groups. The levels of AGP were found to be at least twofold higher which is comparable with the increase in amounts reported after the acute phase response [6]. 

### 3.2. Monosaccharide Analysis

The levels of monosaccharides in the commercially purchased AGP were found (using one-way ANOVA) to be greater (*P* < 0.05) than those in AGP isolated from “normal” heparinised blood. However, direct comparisons were deemed invalid because the methods implemented in the isolation of the glycoprotein differed. It was considered more relevant to use the “normal” blood values as comparisons, having been isolated by the same technique.

**Figure 2:**
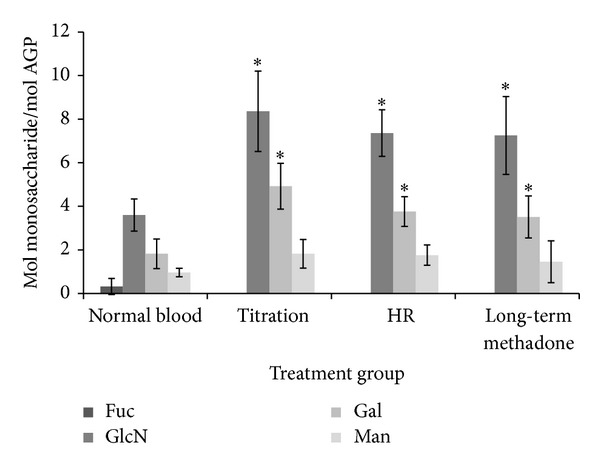
Mean level of monosaccharides in AGP glycans isolated from treatment groups. “Normal blood” represents heparinised blood from the Blood Transfusion Service. *Statistically significant difference compared to normal heparinised blood sample (*P* < 0.05) HR: harm reduction; LT: long-term treatment. Standard deviation calculated from triplicate analysis of a single sample while others were calculated from data from more than one patient (*n* = 4/+).

There was a significant change in the glycosylation of AGP in individuals receiving methadone as an opioid replacement therapy compared to that from a “normal” healthy population. The levels of galactose (Gal) and galactosamine (GlcN; GlcNAc in the *in vivo *structure) were significantly greater in patients undergoing methadone therapy when compared to the “normal” group (refer to Figure 2). Relative consistency was displayed in the level of mannose (Man) between all samples which is consistent with its presence in the complex N-linked oligosaccharide chains of AGP (three mannose residues per chain). The most significant differences in monosaccharide composition occurred with respect to the levels of galactose (Gal) and glucosamine (GlcN) which suggests that there is an increase in the number of branches on the oligosaccharide chains present. AGP has five N-linked chains (so called because they are attached to the side chain of an asparagine amino acid) which can have a variable number of branches attached to a core sequence. Since galactose and the majority of glucosamine (as its acetylated form, N-acetylglucosamine) are only present in the outer branches of N-linked chains, an increase would tentatively suggest that the degree of branching of a chain has also increased. Our data suggests the occurrence of increased branching on the chains of the AGP from the methadone replacement patients. However, in the number of patients analysed, these changes did not appear to be dependent upon stage or types of opioid replacement therapy. The monosaccharide fucose (Fuc), whose presence on oligosaccharide chains is generally correlated with pathophysiological conditions such as inflammation and cancer [18, 19] was not detected in any patient sample.

### 3.3. Oligosaccharide Analysis

**Figure 3:**
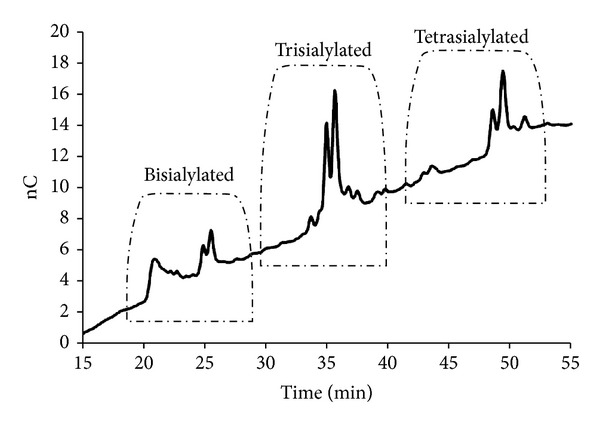
Oligosaccharide library trace generated during separation by HPAEC. Separation based on the charge of oligosaccharide chains as measured using nano-Coulomb (nC). Degree of sialylation highlighted in a dashed line.

Oligosaccharide analysis using HPAEC generated chromatograms which allowed qualitative analysis. The technique initially separates enzymatically cleaved glycans based on the degree of sialylation which provided the main negative charge. Glycans were separated into charge bands, those expressing a single SA residue eluted first (10–20 minutes), followed by bisialylated (20–30 minutes), trisialylated (30–40 minutes), and finally tetrasialylated, eluting between 40 and 50 minutes. An AGP N-linked glycan library was used to allow the corresponding charge groups to be identified (Figure 3). 

The glycosylation of AGP isolated from “normal” heparinised blood samples was found to express numerous structures of different sialylation. Preference in this case appeared to be for bisialylated forms. The traces generated for the oligosaccharides of titration patients indicated that bi- and trisialylated structures were most common although not the only type present. This was similar to the “normal” and commercial samples, implying low levels of branching. Long-term patient AGP was also found to show a greater proportion of bi- and trisialylated structures, however, unlike the titration phase individuals, peaks in the tetrasialylated region were more defined (e.g., LT4, LT9, and LT10). In many patients, the number of peaks was fairly low but they were of a large size suggesting that there were many of the same glycan expressed.

Overall, it was shown that similarities existed between the treatment and nontreatment groups. Titration and “normal” appeared to have a high proportion of trisialylated structures. Variation existed, particularly within the LT group; some AGP displaying more highly branched structures (e.g., LT4 and LT10) while others having a greater proportion of less-sialylated structures (e.g., LT3). 

### 3.4. Drug Binding

The degree of fluorescence quenching increased as the concentration of methadone added was increased, reaching a plateau at concentrations above ~100 *μ*M in both patient AGP and that isolated from “normal” heparinised samples. Values in the absence and presence of 1.07 *μ*M and 250 *μ*M of methadone are recorded in Table 2 to highlight the similarity of participant AGP with that from “normal” hepatrinised blood in the absence of drug. The rate of reduction in fluorescence was most pronounced at low concentrations indicating that binding became saturated relatively quickly. However, the drug did not completely quench the intrinsic fluorescence; some binding sites remained available. In general, the overall reduction in fluorescence was greater for all patient AGP samples analysed when compared to “normal” AGP, however, there were no clear differences between patients. Further studies are required to allow quantification and statistical analysis of binding, and this study was to allow preliminary comparison.

## 4. Conclusion

The success of drug therapies relies on the presence of specific concentrations of its bioactive form (MEC) at the corresponding site of action. Numerous factors determine whether this can be achieved; however, often overlooked is the binding to plasma proteins. Of primary interest to this study was whether the level and glycoform expression of AGP isolated from patients undergoing various stages and types of substitute therapy for opioid dependence differed to a “normal” healthy population. It was supposed that alterations may correlate to variations in the binding of the glycoprotein to methadone. It was hypothesised that AGP isolated from patients would exhibit higher levels and structural changes causing increased binding to methadone when compared to a “normal” AGP sample. Greater binding would at least partially explain why high doses of the drug are required—accounting for that which is bound and therefore inactivated. 

Similar to research undertaken by Rostami-Hodjegan et al. [9], the current study detected increased levels of AGP expressed in individuals who displayed signs of withdrawal from heroin (represented by the titration group). The levels isolated from the patients in the remaining treatment groups—excluding the individual receiving heroin—were also greater than that isolated from the “normal” population. Our study seeks to extend this research by examining the effect of the glycosylation pattern of AGP, and any changes and correlating to the extent of binding to methadone.

Altered glycosylation of AGP has been widely studied in a number of pathophysiological conditions (see reviews in [6, 14]) and compared with the pattern present in healthy subjects which tends towards chains with either two (bi-) or three (tri-) branches. A decrease in the aforementioned number of branches has been demonstrated in acute inflammatory conditions while increased branching is observed to be associated with chronic inflammations, liver diseases, and poor prognosis in cancer after surgery. The data from the current study suggests that AGP isolated from all phases of the methadone treatment programme is associated with a shift towards increased branching. The mixture of isolated glycan chains is separated, by HPAEC, according to the number of terminal negatively charged sialic acids possessed which can normally be directly correlated with the size of the chains. In other words a bi-sialylated structure contains two sialic acid residues which equates to one terminating each of two branches; a tri-sialylated structure has three sialic acids on typically three branches, and a tetra-sialylated chain has four branches each terminated with a sialic acid. Our results indicate that AGP isolated from subjects in the titration and long-term phases both had increases in the tri-sialylated/three branch population, and the latter also contained significant quantities of tetra-sialylated chains with four branches. The significance of these alterations with respect to the drug binding is likely to be related to changes in the conformation of the drug binding site of AGP resulting in either decreased or increased binding affinity for methadone. An increased binding affinity for methadone would result in a larger percentage of the free drug being bound therefore reducing or completely abolishing the desired therapeutic response.

Although it is well understood that structural changes to AGP glycosylation can significantly affect the functions of the biomolecule, there is currently a paucity of research data pertaining to this effect in patients undergoing opioid-replacement therapy. It is well understood that AGP generally exhibits a degree of selectivity for the ligands to which it binds, and, in terms of drugs, they are generally of the neutral or basic variety [8, 14]. If binding to methadone is altered by structural modifications to the glycan chains present on AGP, the free active concentrations and subsequent pharmacological effect could be changed. A reduction in efficacy would become apparent when the affinity increases or vice versa if the affinity was reduced. 

Currently treatment is based on a titration phase where individuals are given low doses of drug to allow for its slow accumulation. Finally, a maintenance dose is determined. However, with investigations like these it may be possible to determine the relative level of glycoforms expressed by an individual's AGP to identify those where it is less likely to be effective or where higher doses will be required. Also, another consequence of i.v heroin use is collapsed veins, complicating venipuncture. However, this preliminary study has shown that potential correlation exists between the structure of AGP glycans and its ability to bind the basic drug methadone although a larger patient population is required to address other influential factors and determine whether the interaction could significantly affect the efficacy of methadone in the treatment of opioid dependencies. 

The variability in the binding of methadone to AGP may partly explain why some individuals require more drug than others to produce the effect. That is not to forget the roles of metabolism and elimination which display interindividual variability due to differences in gene expression. Further work is required to quantify the degree of methadone binding to AGP with specific glycosylation patterns. Fortunately, the ability to quantify the interaction between a plasma protein such as AGP and a drug has recently improved through the use of surface plasma resonance (SPR) [20] which overcomes the limitations of previous methods such as equilibrium dialysis, ultrafiltration, and ultracentrifugation. The latter methods are associated with problems of cost, throughput, and quality of information obtained, as well as being equilibrium based and, therefore, providing no kinetic information. The use of SPR not only overcomes these limitations but also allows the more accurate statistical analysis of the correlation between the degree of binding and drug efficacy.

## Figures and Tables

**Table 1:** Patient demographics.

Stage of therapy	Identity	Age	Male/female	Methadone dose (mg)
Titration	T1	32	F	95
	T2	23	F	65
	T3	37	F	55
	T4	33	M	40
	T5	20	M	60

Harm reduction	HR1	37	F	90
	HR2	28	M	85
	HR3	23	F	150
	HR4	23	F	150

Long term	LT1	35	M	95
	LT2	33	M	150
	LT3	36	F	40
	LT4	44	F	90
	LT5	30	M	105
	LT6	51	M	85
	LT7	34	M	150
	LT8	42	M	50
	LT9	34	M	150
	LT10	31	M	200
	LT11	30	M	90

**Table 2:** Intrinsic fluorescence of AGP in absence and presence of 1.07 *μ*M and 250 *μ*M methadone.

Sample	Fluorescence in the absence of methadone (RFU)	Fluorescence in the presence of 1.07 *μ*M methadone (RFU)	Fluorescence in the presence of 250 *μ*M methadone (RFU)
Commercial (sigma)	41636	38236	30620
“Normal” 1	39943.5	39021	35476
“Normal” 2	37784.5	37032	36138
T1	39859	34006	30712
T2	35992	33371	30840
T3	36715	33020	30284
T4	37571	31089	29182
T5	38882	33959	29412
HR1	39107	33280	29289
HR2	38438	33215	29025
HR3	33531	30023	27072
LT1	31770	30775	27196
LT2	33324	30252	28346
LT4	38688	37189	31008
LT5	39937	36241	31893
LT6	39691	31681	27137
LT7	38484	31538	28658

## References

[1] UNODC (2010). *World Drug Report 2010*.

[2] McKeganey N, Bloor M, Robertson M, Neale J, MacDougall J (2006). Abstinence and drug abuse treatment: results from the drug outcome research in Scotland Study. *Drugs*.

[3] Romach MK, Piafsky KM, Abel JG (1981). Methadone binding to orosomucoid (*α*1-acid glycoprotein): determinant of free fraction in plasma. *Clinical Pharmacology and Therapeutics*.

[4] Drummer O, Odell M (2001). *The Forensic Pharmacology Drugs of Abuse*.

[5] Scimeca MM, Savage SR, Portenoy R, Lowinson J (2000). Treatment of pain in methadone-maintained patients. *Mount Sinai Journal of Medicine*.

[6] Ceciliani F, Pocacqua V (2007). The acute phase protein *α*1-acid glycoprotein: a model for altered glycosylation during diseases. *Current Protein and Peptide Science*.

[7] Kuroda Y, Matsumoto S, Shibukawa A, Nakagawa T (2003). Capillary electrophoretic study on pH dependence of enantioselective disopyramide binding to genetic variants of human *α*1-acid glycoprotein. *Analyst*.

[8] Kremer JMH, Wilting J, Janssen LHM (1988). Drug binding to human alpha-1-acid glycoprotein in health and disease. *Pharmacological Reviews*.

[9] Rostami-Hodjegan A, Wolff K, Hay AWM, Raistrick D, Calvert R, Tucker GT (1999). Population pharmacokinetics of methadone in opiate users: characterization of time-dependent changes. *British Journal of Clinical Pharmacology*.

[10] Varki A, Cummings RD, Esko JD (2009). *Essentials of Glycobiology*.

[11] Taylor ME, Drickamer K (2003). *Introduction to Glycobiology*.

[12] van Dijk W, Turner GA, Mackiewicz A (1994). Changes in glycosylation of acute-phase proteins in health and disease: occurrence, regulation and function. *Glycosylation and Disease*.

[13] Higai K, Aoki Y, Azuma Y, Matsumoto K (2005). Glycosylation of site-specific glycans of *α*1-acid glycoprotein and alterations in acute and chronic inflammation. *Biochimica et Biophysica Acta*.

[14] Israili ZH, Dayton PG (2001). Human alpha-1-glycoprotein and its interactions with drugs. *Drug Metabolism Reviews*.

[15] Muller WE, Wainer IW, Drayer DE (1988). Stereoselective plasma protein binding of drugs. *Drug Stereochemistry: Analytical Methods and Pharmacology*.

[16] Elliott MA, Elliott HG, Gallagher K, McGuire J, Field M, Smith KD (1997). Investigation into the concanavalin A reactivity, fucosylation and oligosaccharide microheterogeneity of *α*1-acid glycoprotein expressed in the sera of patients with rheumatoid arthritis. *Journal of Chromatography B*.

[17] Fan J-Q, Namiki Y, Matsuoka K, Lee YC (1994). Comparison of acid hydrolytic conditions for Asn-linked oligosaccharides. *Analytical Biochemistry*.

[18] Turner GA, Skillen AW, Buamah P (1985). Relation between raised concentrations of fucose, sialic acid, and acute phase proteins in serum from patients with cancer: choosing suitable serum glycoprotein markers. *Journal of Clinical Pathology*.

[19] Listinsky JJ, Siegal GP, Listinsky CM (1998). Alpha-L-fucose: a potentially critical molecule in pathologic processes including neoplasia. *American Journal of Clinical Pathology*.

[20] Gustafsson SS, Vrang L, Terelius Y, Danielson UH (2011). Quantification of interactions between drug leads and serum proteins by use of ‘binding efficiency’. *Analytical Biochemistry*.

